# Comprehensive genomic characterization of gene therapy-induced T-cell acute lymphoblastic leukemia

**DOI:** 10.1038/s41375-020-0779-z

**Published:** 2020-03-03

**Authors:** Peter Horak, Sebastian Uhrig, Maximilian Witzel, Irene Gil-Farina, Barbara Hutter, Tim Rath, Laura Gieldon, Gnana Prakash Balasubramanian, Xavier Pastor, Christoph E. Heilig, Daniela Richter, Evelin Schröck, Claudia R. Ball, Benedikt Brors, Christian J. Braun, Michael H. Albert, Claudia Scholl, Christof von Kalle, Manfred Schmidt, Stefan Fröhling, Christoph Klein, Hanno Glimm

**Affiliations:** 1 German Cancer Consortium (DKTK), Heidelberg, Germany; 2 Division of Applied Bioinformatics, German Cancer Research Center (DKFZ) and National Center for Tumor Diseases (NCT) Heidelberg, Heidelberg, Germany; 3 Faculty of Biosciences, Heidelberg University, Heidelberg, Germany; 4 Department of Pediatrics, Dr. von Hauner Children’s Hospital, Ludwig Maximilians University, Munich, Germany; 5 Division of Translational Oncology, DKFZ and NCT Heidelberg, Heidelberg, Germany; 6 GeneWerk GmbH, Heidelberg, Germany; 7 Institute for Clinical Genetics, Medical Faculty Carl Gustav Carus, Technische Universität Dresden, Dresden, Germany; 8 German Cancer Consortium (DKTK) Dresden and DKFZ, Heidelberg, Germany; 9 NCT Dresden, Dresden, Germany; 10 Division of Pediatric Neurooncology, DKFZ, Heidelberg, Germany; 11 Division of Theoretical Bioinformatics, DKFZ, Heidelberg, Germany; 12 Heidelberg Center for Personalized Oncology (DKFZ-HIPO), Heidelberg, Germany; 13 Department of Translational Medical Oncology, NCT Dresden, Dresden, Germany; 14 University Hospital Carl Gustav Carus, Technische Universität Dresden, Dresden, Germany; 15 Division of Applied Functional Genomics, DKFZ, Heidelberg, Germany; 16 Division of Translational Medical Oncology, DKFZ and NCT Heidelberg, Heidelberg, Germany

**Keywords:** Translational research, Cancer genomics

## To the Editor:

Ten pediatric patients with severe Wiskott–Aldrich syndrome (WAS) have received autologous bone marrow transplantation of gene-corrected CD34+ cells within a hematopoietic stem cell gene therapy (GT) trial [1]. Following GT, nine out of ten treated patients had nearly complete remission of their clinical disease and showed sustained expression of WAS protein in myeloid and lymphoid cells. However, 1–5 years later, they developed T-cell acute lymphoblastic leukemia (T-ALL) and/or acute myeloid leukemia. The clinical course of these patients and characterization of the retroviral integration sites within the leukemic clones have been reported [2]. All six T-ALL clones harbored retroviral vector integrations within or close to the *LMO2* gene locus and one to eight additional integration sites, including integrations upstream of T-ALL proto-oncogenes *TAL1* (WAS5 and WAS7) and *LYL1* (WAS8 and WAS10). LMO2, a transcriptional regulator of hematopoiesis, is continuously downregulated during development and repressed in mature T cells. LMO2 overexpression maintains a pool of preleukemic cells that subsequently acquire further oncogenic mutations [3]. Despite much progress in understanding the role of LMO2 in T-ALL, the functional or spatiotemporal role of secondary genetic driver events remains unclear. Given the circumstances of this study, in which the time of the primary genetic event leading to LMO2 overexpression in hematopoietic stem or progenitor cells is known, we anticipated insights into secondary genetic alterations leading to leukemogenesis and performed comprehensive genomic and transcriptomic profiling of T-ALL cases driven by retroviral insertional mutagenesis of LMO2.

Immunophenotyping classified three T-ALL cases (WAS1, WAS5, WAS8) as double positive (CD4+CD8+) and WAS10 as CD8 single positive, corresponding to later developmental stages of thymocytes. WAS6 and WAS7 were CD4/CD8 negative, with dim CD5 expression and in case of WAS6 missing CD1 expression, suggesting the phenotype of early T-cell precursor (ETP) ALL [4]. RNA sequencing (RNA-Seq) confirmed the differences in CD4 and CD8 expression. WAS6 and WAS7 both lacked CD1A RNA expression and demonstrated elevated RNA expression of CD117 (Supplementary Table 1).

**Fig. 1:**
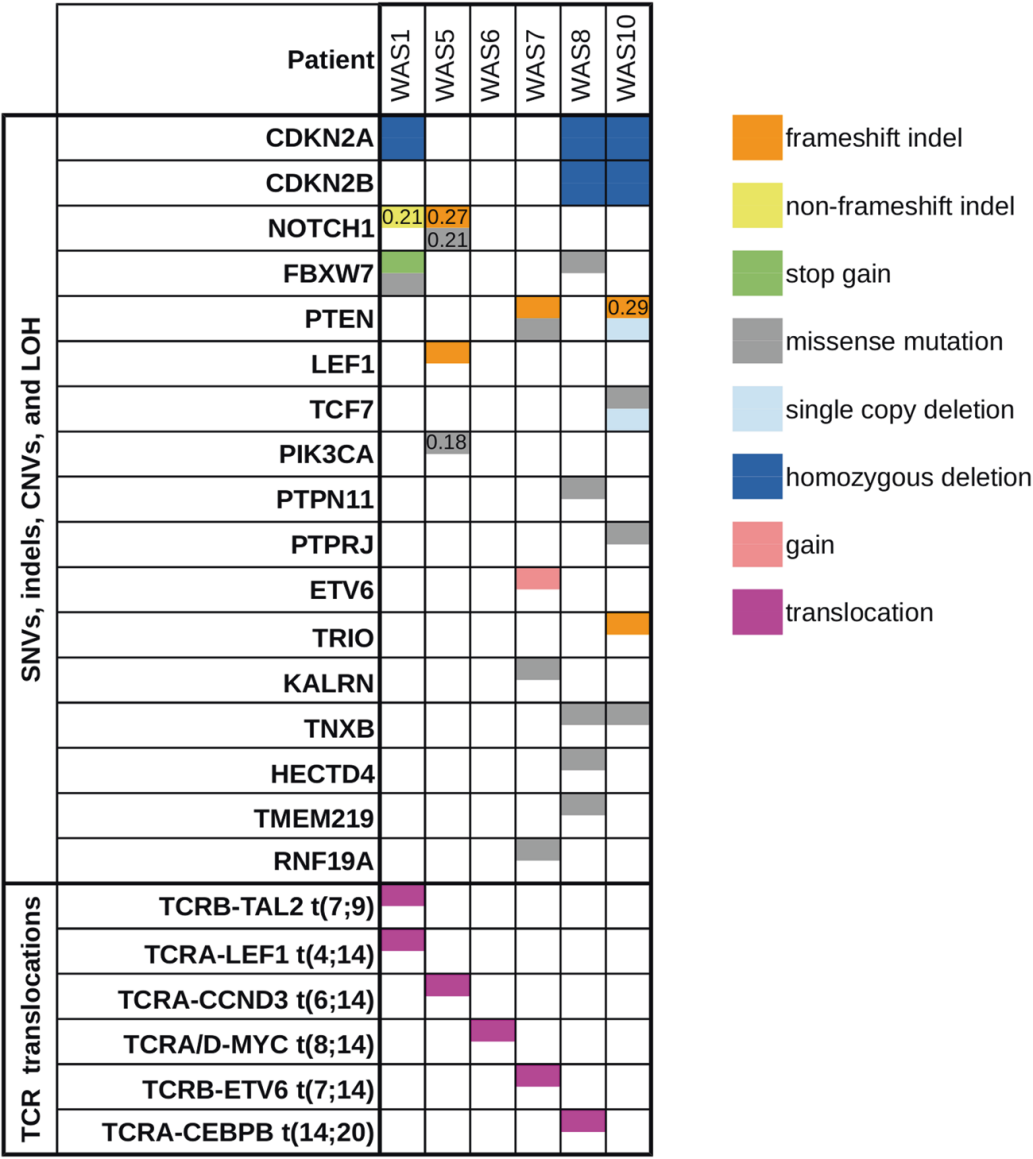
OncoPrint visualization of relevant molecular alterations in gene therapy-induced T-ALL patients. Half-colored table cells indicate events in a single allele, fully colored table cells indicate biallelic events. MAFs for subclonal events are shown.

**Fig. 2:**
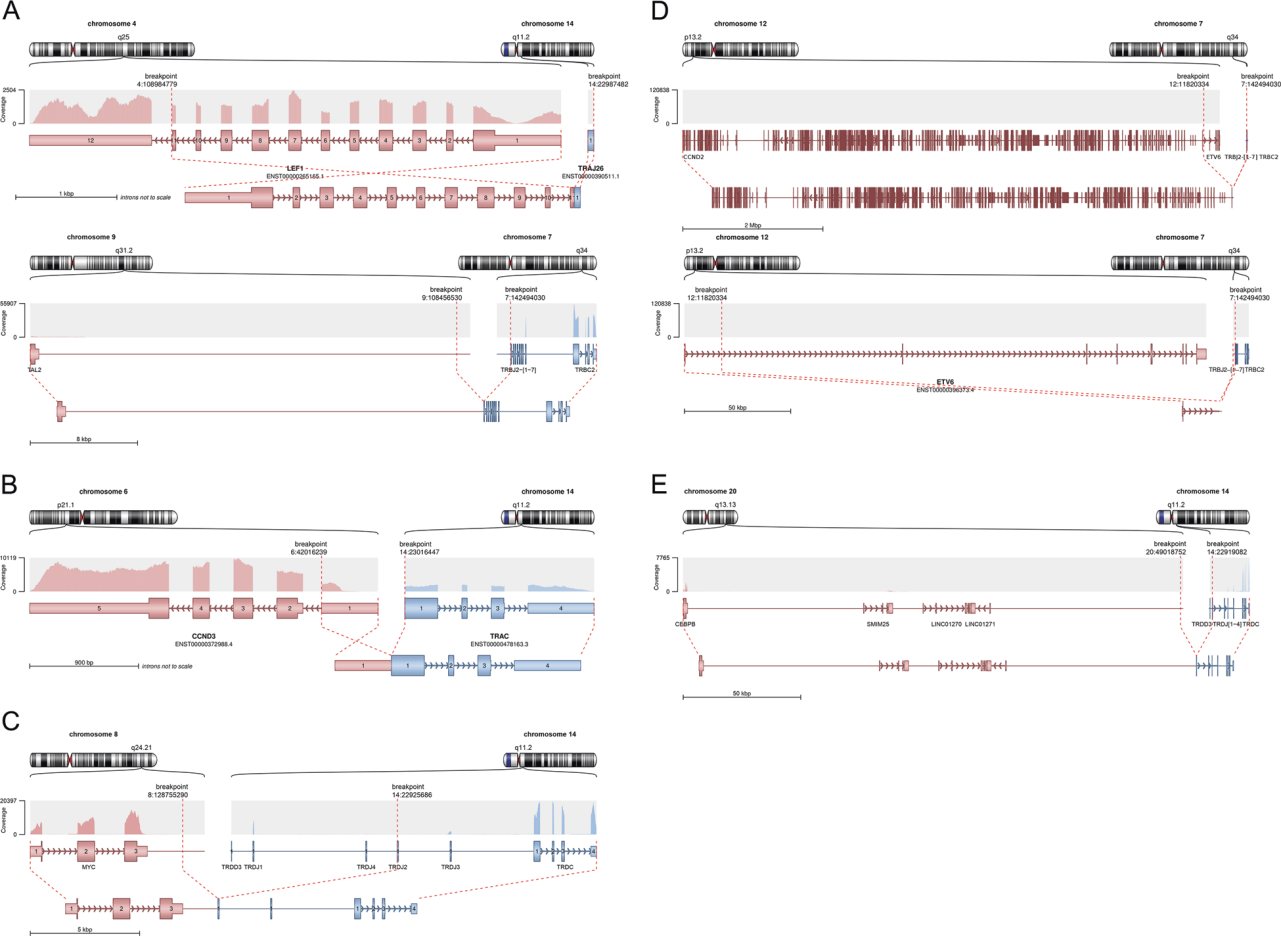
Structure of translocations and gene fusions. Exon structure and coverage of exons in transcriptome sequencing data are shown. Fusion breakpoints are indicated by red dashed lines. **a** Oncogenic *TAL2-TCRB* fusion and translocation t(4;14) resulting in a *LEF1-TCRA/D* in WAS1. **b** Translocation t(6;14) leading to *CCND3-TCRA/D* fusion in WAS5. **c** *MYC-TCRA/D* fusion in WAS6. **d** Translocation of the *TCRB* locus to a breakpoint on chromosome 12 approximately 7 Mbp downstream of *CCND2* (upper panels) and within intron 1 of the *ETV6* gene (lower panels) in WAS7. **e** Breakpoint region on chromosome 20q13.13 in WAS8 is localized ~200 kbp downstream of *CEBPB*.

Whole genome sequencing (WGS) identified secondary single-nucleotide variants (SNVs) and small insertions/deletions (indels) at frequencies ranging from one somatic SNV and one indel in WAS6 to 18 SNVs and two indels in WAS10, many of them in previously described T-ALL driver genes (Fig. 1, Supplementary Fig. 1a). Cumulative number of SNVs and indels showed a linear increase over time from GT to T-ALL diagnosis (Supplementary Fig. 1b). Some of the secondary molecular alterations were subclonal, with a mutant allele fraction (MAF, corrected for leukemic cell purity) of less than 0.3 (Supplementary Fig. 1a and Supplementary Table 2). Mutations of the *NOTCH1* heterodimerization domain were found in WAS1 and WAS5, with WAS5 harboring an additional PEST domain mutation. WAS1 also displayed two different *FBXW7* mutations. WAS8 displayed a homozygous *FBXW7* missense mutation (corrected MAF 0.92). PI3K/AKT signaling was affected by loss-of-function *PTEN* mutations (WAS7, WAS10) or a *PI3KCA* mutation (WAS5). An oncogenic *PTPN11* (*SHP-2*) mutation led to RAS/ERK pathway activation in WAS8 (Fig. 1). In addition to the potentially inactivating *LEF1*-*TCRA/D* gene fusion in WAS1 (Fig. 2a), a frameshift mutation in *LEF1* was detected in WAS5. WAS10 also harbored a frameshift insertion in the gene encoding the Rho guanine nucleotide exchange factor (RhoGEF) TRIO, a novel *TCF7* missense mutation, and a missense mutation in *PTPRJ* (protein tyrosine phosphatase involved in FLT3 regulation).

Insertional mutagenesis including the involvement of a dominant LMO2 clone was demonstrated in all cases and T-cell receptor rearrangements were reported in five patients [2]. We can confirm most of the cytogenetically detected translocations and gene fusions. In WAS1, the oncogenic *TAL2-TCRB* fusion and a novel translocation t(4;14) resulting in a *LEF1-TCRA/D* fusion were detected by RNA-Seq (Fig. 2a). In WAS5, the previously reported translocation t(1;8) (q31;q23) could not be identified by WGS or RNA-Seq, yet a translocation t(6;14) leading to *CCND3-TCRA/D* gene fusion was present (Fig. 2b). The *MYC-TCRA/D* fusion in WAS6 led to the highest *MYC* expression in our cohort (Fig. 2c). WAS7 has been described to harbor a translocation t(7;12) resulting in a *TCRB-CCND2* fusion. While we detected fusion reads between chromosomal locations on chromosomes 7q and 12p, the breakpoint on chromosome 12 was more than 7 Mbp downstream of the *CCND2* locus and did not lead to elevated *CCND2* RNA expression. We identified a possible translocation of the *TCRB* locus to intron 1 of the *ETV6* gene (Fig. 2d) in WAS7. A *CEBPB-TCRA/D* fusion, analogous to an oncogenic rearrangement reported in B-ALL [5], was reported in WAS8. WGS-identified breakpoint on chromosome 20q13.13 was localized in an intergenic region ~200 kbp downstream of the *CEBPB* transcription start (Fig. 2e) suggesting another pathogenic mechanism such as indirect regulation by enhancer hijacking leading to elevated *CEBPB* RNA expression.

Biallelic deletions at 9p21 targeting the *CDKN2A/CDKN2B* locus were observed in WAS1, WAS8, and WAS10 (Fig. 1). Loss of heterozygosity (LOH) on the short arm of chromosome 9 was identified in WAS1, WAS5, WAS8, and WAS10, indicating a copy number neutral LOH of the *CDKN2A/CDKN2B* locus in WAS5 (Supplementary Fig. 2b). Duplication of chromosome 17q, usually associated with hyperdiploid pediatric B-cell precursor ALL, was demonstrated in WAS1 and WAS5 (Supplementary Fig. 2a). Transcriptome analysis of WAS1 and WAS5 revealed recurrently overexpressed genes within the region on chromosome 17q, specifically *CBX1*, *CBX2*, *FASN*, *PRKCA*, *STAT5A*, and *TK1*. Copy number aberrations on chromosome 6q, as observed in WAS6, are frequent events in T-ALL and this region encompasses multiple tumor suppressor gene candidates, including *BACH2*, *CCNC*, *CASP8AP2*, and *EPHA7*.

We assessed global gene expression profiles of five WAS cases by RNA-Seq (WAS1, WAS5, WAS6, WAS7, and WAS8) at the time of T-ALL diagnosis and compared them with published expression profiles of 264 pediatric acute lymphoblastic leukemia samples [6]. Supervised hierarchical clustering based on differentially expressed marker genes of eight defined molecular subgroups (Supplementary Table 3) led to grouping of WAS1, WAS5, WAS7, and WAS8 cases with the clusters of TAL1 and TAL2 driven T-ALL (Supplementary Fig. 3a). WAS6, on the other hand, associated most significantly with the LMO2/LYL1 cluster (Supplementary Fig. 3a and Supplementary Table 4). When LMO2 expression was included in this analysis, all GT-induced T-ALL cases clustered with TAL1/2 and LMO2/LYL1 cases (Supplementary Fig. 3b and Supplementary Table 4). Double-positive and CD8-positive T-ALL cases show recurrent alterations of cell cycle regulators (4/4) and mutations in NOTCH signaling pathway (3/4), transcriptional regulators of the WNT pathway (3/4) as well as PI3K or RAS signaling (3/4). ETP-like, double-negative cases presented with a smaller number of genetic lesions and lack of clearly defined or recurrent secondary alterations. Other recurrently mutated or otherwise altered pathways were linked to ubiquitination (*RNF19A*, *HECTD4, USP7*), epigenetic regulation, and chromatin remodeling (*CBX1*, *CBX2*, *CREBBP*, *MYT1L*). We observed singular alterations in transcription factor *PAX5*, serine/threonine kinase *LMTK3*, and RhoGEF *KALRN* (Fig. 1). We analyzed mRNA expression of ten genes most frequently affected by retroviral integration in each leukemic clone (Supplementary Table 5) [2]. Presence of retroviral integration sites did not correlate with deregulated RNA expression of affected genes. We were unable to identify any novel integration sites based on WGS data, except for peripheral blood mononuclear cells from all patients but WAS10 harboring a putative integration of the Torque Teno virus. Mutational signature analysis did not reveal substantial differences between samples (Supplementary Fig. 4).

T-ALL development after GT is driven by retroviral *LMO2* integration and orchestrated by a variety of secondary molecular alterations, including individual oncogenic translocations [2, 7, 8]. The higher frequency of leukemia in the WAS cohort in comparison to other hematopoietic stem cell GT studies might be influenced by the specific vector system, underlying disease biology (cf. ADA-SCID) [9], higher vector copy numbers used for the transduction of hematopoietic stem cells, or other as yet unknown factors [2, 10, 11]. Leukemic blasts in peripheral blood already harbored subclonal secondary mutations at the time of diagnosis, thus preventing a longitudinal study of secondary alterations, which were presumably acquired in the thymic niche.

While LMO2 alterations are seen in a wide context of genetic subtypes and at several developmental stages, LMO2 expression is characteristic for a subset of TAL1/2 as well as LYL1-driven leukemia while simultaneous LMO2/LYL1 rearrangements define a specific subset of pediatric T-ALL clustering with TAL-LMO rearranged cases [12]. Our data support the hypothesis that retrovirally induced LMO2-driven T-ALL phenotypically resembles the TAL/LMO subtype of sporadic T-ALL. LMO2 overexpression in murine models induces thymocyte self-renewal rather than outright leukemia [3]. Gene expression profiling of CD2-Lmo2-driven mouse models suggested their resemblance to LYL1-driven human T-ALL [3] and differed from observations in murine T-ALL caused by insertional mutagenesis in AKXD mice [13], which co-express Tal1 and resemble more closely human T-ALL developing in GT patients. To resolve the question which model faithfully represents human LMO2-driven leukemogenesis, a mouse model transplanted with LMO2-transduced human hematopoietic stem cells showed accumulation of double-positive CD3− cells with a subset displaying early developmental block at the double-negative stage [14]. This observation is mirrored in our cohort, with half of the patients presenting with CD4+CD8+CD3− T-ALL. Transcriptomic signatures of the double-positive LMO2-transduced human hematopoietic stem cells cluster together with the TAL/LMO signature [15], and correlate with the clustering of the WAS cases between the TAL1/2 and LMO2/LYL1 subgroup. WAS6 and WAS7 display a double-negative phenotype and show some similarities to the ETP-like T-ALL subgroup, even though WAS7 displayed CD1 expression along with a retroviral insertion site in TAL1 and PTEN mutations, which are characteristic of the TAL1 subtype.

Although we are able to define common altered pathways and infer genotypic and phenotypic characteristics of GT-associated T-ALL, some limitations of our study remain. They include the shortcoming of current next-generation sequencing methods toward complex structural variants and rearrangements. Our data identify oncogenic translocations as primary driving lesions, though functional consequences of rare *ETV6* or *CEBPB* translocations in T-ALL are yet unclear. In addition, unavailability of sufficient material from preleukemic samples prevented the deciphering of the sequence of secondary events.

Our results support the hypothesis of acquisition of secondary mutations over time in GT-induced T-ALL. We describe unifying features of GT-induced T-ALL, such as expression profiles resembling TAL1/2 and LMO2/LYL1-driven T-ALL and development of CD4+CD8+CD3− disease in half of the cases. We propose that similar mechanisms drive sporadic and retrovirally driven leukemogenesis in human thymocytes.

## Supplementary information

Supplementary Information

Supplemental Table 1

Supplemental Table 2

Supplemental Table 3

Supplemental Table 4

Supplemental Table 5

Supplemental Figure 1

Supplemental Figure 2

Supplemental Figure 3

Supplemental Figure 4

## Data Availability

The raw analytical data were deposited in the European Genome-phenome Archive (https://www.ebi.ac.uk/ega/datasets) under the accession number EGAS00001003870.
