# Publisher Correction: Comprehensive genomic characterization of gene therapy-induced T-cell acute lymphoblastic leukemia

**DOI:** 10.1038/s41375-021-01364-5

**Published:** 2021-11-16

**Authors:** Peter Horak, Sebastian Uhrig, Maximilian Witzel, Irene Gil-Farina, Barbara Hutter, Tim Rath, Laura Gieldon, Gnana Prakash Balasubramanian, Xavier Pastor, Christoph E. Heilig, Daniela Richter, Evelin Schröck, Claudia R. Ball, Benedikt Brors, Christian J. Braun, Michael H. Albert, Claudia Scholl, Christof von Kalle, Manfred Schmidt, Stefan Fröhling, Christoph Klein, Hanno Glimm

**Affiliations:** 1 German Cancer Consortium (DKTK), Heidelberg, Germany; 2 Division of Applied Bioinformatics, German Cancer Research Center (DKFZ) and National Center for Tumor Diseases (NCT) Heidelberg, Heidelberg, Germany; 3 Faculty of Biosciences, Heidelberg University, Heidelberg, Germany; 4 Department of Pediatrics, Dr. von Hauner Children’s Hospital, Ludwig Maximilians University, Munich, Germany; 5 Division of Translational Oncology, DKFZ and NCT Heidelberg, Heidelberg, Germany; 6 GeneWerk GmbH, Heidelberg, Germany; 7 Institute for Clinical Genetics, Medical Faculty Carl Gustav Carus, Technische Universität Dresden, Dresden, Germany; 8 German Cancer Consortium (DKTK) Dresden and DKFZ, Heidelberg, Germany; 9 NCT Dresden, Dresden, Germany; 10 Division of Pediatric Neurooncology, DKFZ, Heidelberg, Germany; 11 Division of Theoretical Bioinformatics, DKFZ, Heidelberg, Germany; 12 Heidelberg Center for Personalized Oncology (DKFZ-HIPO), Heidelberg, Germany; 13 Department of Translational Medical Oncology, NCT Dresden, Dresden, Germany; 14 University Hospital Carl Gustav Carus, Technische Universität Dresden, Dresden, Germany; 15 Division of Applied Functional Genomics, DKFZ, Heidelberg, Germany; 16 Division of Translational Medical Oncology, DKFZ and NCT Heidelberg, Heidelberg, Germany

**Keywords:** Translational research, Cancer genomics

Correction to: *Leukemia* 10.1038/s41375-020-0779-z, published online 03 March 2020

The article Comprehensive genomic characterization of gene therapy-induced T-cell acute lymphoblastic leukemia, written by Peter Horak, Sebastian Uhrig, Maximilian Witzel, Irene Gil-Farina, Barbara Hutter, Tim Rath, Laura Gieldon, Gnana Prakash Balasubramanian, Xavier Pastor, Christoph E. Heilig, Daniela Richter, Evelin Schröck, Claudia R. Ball, Benedikt Brors, Christian J. Braun, Michael H. Albert, Claudia Scholl, Christof von Kalle, Manfred Schmidt, Stefan Fröhling, Christoph Klein & Hanno Glimm, was originally published Online First without Open Access. After publication in volume 34, pages 2785–2789, the author decided to opt for Open Choice and to make the article an Open Access publication. Therefore, the copyright of the article has been changed to © The Author(s) [year of online publication] and the article is forthwith distributed under the terms of the Creative Commons Attribution.

