# Supplementary material for: Comprehensive genomic characterization of gene therapy-induced T-cell acute lymphoblastic leukemia
Source: Leukemia. 2020 Mar 3;34(10):2785–9. doi: 10.1038/s41375-020-0779-z (PMC8321895; doi:10.1038/s41375-020-0779-z)
Supplement: Supplementary file 1 — Supplementary Information [file 41375_2020_779_MOESM1_ESM.docx]

Supplementary information

Comprehensive Genomic Characterization of Gene Therapy-Induced T-cell Acute Lymphoblastic Leukemia

Peter Horak,^1,2,#^ Sebastian Uhrig,^2-4^ , Maximilian Witzel,^5^ Irene Gil-Farina,^6^ Barbara Hutter,^2,3^ Tim Rath,^6,16^ Laura Gieldon,^7-9^ Gnana Prakash Balasubramanian,^3,10^ Xavier Pastor,^11,12^ Christoph E. Heilig,^1,2^ Daniela Richter,^9,13,14^ Evelin Schröck,^7-9^ Claudia R. Ball,^9,13,14^ Benedikt Brors,^2,3^ Christian J. Braun,^5^ Michael H. Albert,^5^ Claudia Scholl,^15^ Christof von Kalle,^6,16^ Manfred Schmidt,^6,16^ Stefan Fröhling,^1,2^ Christoph Klein,^5^ Hanno Glimm,^9,13,14,#^

1. Department of Translational Medical Oncology, National Center for Tumor Diseases (NCT) Heidelberg and German Cancer Research Center (DKFZ), Heidelberg, Germany
2. German Cancer Consortium (DKTK), Heidelberg, Germany
3. Division of Applied Bioinformatics, DKFZ and NCT Heidelberg, Heidelberg, Germany
4. Faculty of Biosciences, Heidelberg University, Heidelberg, Germany
5. Department of Pediatrics, Dr. von Hauner Children’s Hospital, Ludwig Maximilians University, Munich, Germany
6. Department of Translational Oncology, National Center for Tumor Diseases (NCT) Heidelberg and German Cancer Research Center (DKFZ), Heidelberg, Germany
7. Institute for Clinical Genetics, Medical Faculty Carl Gustav Carus, Technische Universität Dresden, Dresden, Germany
8. German Cancer Consortium (DKTK) Dresden and German Cancer Research Center (DKFZ), Heidelberg, Germany
9. NCT Dresden, Dresden, Germany
10. Division of Pediatric Neurooncology, DKFZ, Heidelberg, Germany
11. Division of Theoretical Bioinformatics, DKFZ, Heidelberg, Germany
12. Heidelberg Center for Personalized Oncology (DKFZ-HIPO), Heidelberg, Germany
13. Department of Translational Medical Oncology, NCT Dresden, Dresden, Germany
14. University Hospital Carl Gustav Carus, Technische Universität Dresden, Dresden, Germany
15. Division of Applied Functional Genomics, DKFZ, Heidelberg, Germany
16. GeneWerk GmbH, Heidelberg, Germany

Supplemental Tables: 5

Supplemental Figures: 4

# Supplemental Table Legends

**Supplemental Table 1.** Immunophenotype of GT-induced T-ALL as defined by flow cytometry contrasted with expression data showing DESeq normalized counts of specific immunophenotypic markers of T-ALL as well as relative variation within the cohort.

*WAS1 and WAS8 also developed AML; BM: bone marrow; PB: peripheral blood; PB-frozen: immunophenotyping performed on thawed PBMC

**Supplemental Table 2.** Annotated output of the bioinformatic analysis showing all detected SNVs, germline SNVs, Indels, germline Indels, deletions, duplications, inversions, translocations and copy number alterations as detected by the respective variant calling algorithm. Leukemia cell content was determined from WGS data using purity estimates by the ACESeq algorithm. MAFs corrected for purity are reported together with raw values. Sequencing coverage metrics of whole-genome and RNA-sequencing.

**Supplemental Table 3.** Differentially expressed marker genes of each of the eight defined molecular subgroups[^1^](#_ENREF_1) used for the supervised hierarchical clustering.

**Supplemental Table 4.** Detailed molecular characteristics of pediatric T-ALL cases co-clustering with GT-induced T-ALL as identified by hierarchical clustering with and without considering LMO2 expression.

**Supplemental Table 5.** Expression of 10 genes most frequently affected by retroviral integration in each leukemic clone showing relative expression values within the cohort.

# Supplemental Figure Legends

**Supplemental Figure 1.**(A) Absolute count of single nucleotide variants (SNVs) and small insertions/deletions (Indels) and the percentage of subclonal SNVs and Indels (MAF<0.3) in GT-induced T-ALL patients. Age (in years) and days after gene therapy at ALL diagnosis are shown for each individual patient (B) Correlation of the combined number of functional SNVs/Indels (upper graph) and all SNVs/Indels (lower graph) on y-axis and days after gene therapy on x-axis. Dots are annotated by WAS patient number.

**Supplemental Figure 2.** Copy number plots (A) and B-allele frequency plots (BAF) (B) in gene therapy-induced T-ALL. (A) Red arrows point to biallelic deletions of 9p21 in WAS1, WAS8 and WAS10. Green regions depict chromosomal copy number gains, in particular duplications on chromosome 17q in WAS1 and WAS5. Red regions show copy number losses. (B) LOH of chromosome 9p is seen in WAS1, WAS5, WAS8 and WAS10.

**Supplemental Figure 3.**(A) Supervised hierarchical clustering of global gene expression profiles of 264 pediatric T-ALL cases together with five GT-induced T-ALL cases based on differentially expressed marker genes for eight distinct transcriptomic subgroups including a heatmap of marker gene expression (B) Same hierarchical clustering analysis and heatmap additionally including LMO2 expression as marker for the LMO2/LYL1 and LMO1/2 cluster. GT-induced T-ALL cases are marked in green.

**Supplemental Figure 4.**(A) Contribution of mutational signatures according to Alexandrov-COSMIC (AC) signatures and (B) mutational signatures identified in a cohort of pediatric leukemias and solid tumors with corresponding AC signatures in parentheses. (C) Individual mutational spectra of single base-pair substitutions in GT-induced T-ALL.

# Supplemental Materials and Methods

## Material resources

This study includes genomic and transcriptomic data acquired from 6 WAS patients, ranging between 2 to 14 years of age that underwent gene therapy with γ-retrovirally transduced hematopoietic stem cells.[^2^](#_ENREF_2)^,^ [^3^](#_ENREF_3) The according trial is registered in the German Clinical Trials Register (DRKS00000330), and the clinical GT protocol was reviewed and approved by the Ethics Committees of Hannover Medical School and Ludwig Maximilians University Munich. All procedures were performed following the Declaration of Helsinki Guidelines and under the strict observation of the German regulatory authority for biologics/cell therapies (Paul Ehrlich Institute).

## Next-generation sequencing, bioinformatic analysis and target validation

DNA from leukemic cells and normal PBMCs was isolated using the AllPrep DNA/RNA/miRNA Universal Kit (Qiagen), followed by quality control using gel electrophoresis and a TapeStation 2200 system (Agilent). From each of the samples from WAS1/5/8 we extracted 1 µg of genomic DNA, which was fragmented to 250 bp insert size with a Covaris S2 device and libraries were prepared using the TruSeq DNA LT Sample Prep Kit (Illumina). The final libraries were validated using a Qubit 2.0 Fluorometer (Life Technologies) and a Bioanalyzer 2100 system (Agilent), before sequencing on the HiSeq 2000 v3 platform (Illumina). The DNA libraries from WAS6 were sequenced alike, but the details of the library preparation could not be reconstructed due to missing information. 1 µg of genomic DNA from each of the WAS7 samples was fragmented to 350 bp insert size with a Covaris S2 device. Libraries were prepared using the Next Ultra DNA Library Prep Kit (NEB) and validated as described above. Sequencing was carried out on the HiSeq 2000 v3 and HiSeq 2500 v3 platforms. 100 ng of genomic DNA from each of the WAS10 samples was fragmented to 450 bp insert size with a Covaris LE220 device and libraries were prepared using the TruSeq Nano LT DNA Sample Prep Kit (Illumina). The final libraries were validated using a Qubit Assay on a FilterMax F3 Multi-Mode Microplate Reader (Molecular Devices) and a TapeStation 4200 system, before sequencing on the HiSeq X platform. Using the TruSeq RNA Sample Preparation Kit (Illumina), cDNA libraries were prepared from 1 µg of total RNA sheared to fragments of 150 bp. The quality of the cDNA libraries was assessed with the help of a Qubit 2.0 Fluorometer and a Bioanalyzer 2100 system. For all libraries paired-end sequencing was carried out according to the manufacturer's recommendations, yielding read lengths of 101 bp (HiSeq 2000/2500) or 151 bp (HiSeq X).

Reads from DNA- and RNA-Seq were mapped to the 1000 Genomes Phase II assembly of the human reference genome (NCBI build 37.1) as described before.[^4^](#_ENREF_4) Sequencing coverage metrics are shown in Supplemental Table 2. Single-nucleotide variants (SNVs) and small insertions/deletions (indels) were analyzed using a previously reported bioinformatics workflow.[^4^](#_ENREF_4) We used ACEseq[^5^](#_ENREF_5) to identify copy number aberrations (CNA) and loss of heterozygosity (LOH) as well as tumor cell content (TCC) and ploidy of a sample. For TCC estimation, values in the range of 0.15-1.0 were tested, and a ploidy range of 1.0-6.5 was allowed. For each possible combination of TCC and ploidy, absolute and allele-specific copy numbers and the decrease in heterozygosity (DH) were estimated segment-wise. Allele-specific copy numbers were calculated as total copy number (TCN) divided by two for balanced segments and as a function of coverage and B allele read counts in case of imbalanced segments. The weighted mean distance of all segments to the next allowed integer copy number state was calculated for total and allele-specific copy numbers, where allowed means even TCN states for balanced segments and any integer copy number state for imbalanced segments and allele-specific copy numbers. TCC/ploidy combinations requiring negative copy number states or a DH > 1 for any segment were excluded. Local minima in the weighted mean distance were considered as possible TCC/ploidy solution for the sample and were visually evaluated. Additionally, TCC was estimated from the mutant allele fraction distribution of somatic SNVs, and CNA- and SNV-based estimates were compared. To select CNAs, segments with a TCN at least 0.7 above the selected ploidy were defined as gains and segments with a TCN at least 0.3 below it were definded as losses. Segments with LOH, including copy number neutral ones, were also reported. Segments not fitting to an integer TCN were considered subclonal CNAs. Structural variants were detected using CREST.[^6^](#_ENREF_6) Gene fusions were extracted from the RNA-Seq data with the help of Arriba (https://github.com/suhrig/arriba/). ACEseq and CREST events were annotated with RefSeq genes using BEDTools[^7^](#_ENREF_7). The contributions of mutational signatures were calculated by the R/Bioconductor package YAPSA using the Alexandrov-COSMIC signatures (<https://cancer.sanger.ac.uk/cosmic/signatures_v2>)[^8^](#_ENREF_8) as well as signatures from Ma et al.[^9^](#_ENREF_9)

We developed *D-ViSioN* (Detection of Integration of Virus(s) by SingletoNs) procedure to predict putative viral integration events. Reads from WGS and RNA-Seq were mapped by Burrows-Wheeler Aligner (version 0.5.10) to the viral genome collection from NCBI (<ftp://ftp.ncbi.nih.gov/refseq/release/viral/viral.1.1.genomic.fna>). Mapped reads and their unmapped mates were subsequently aligned to the human reference genome (hg19, UCSC) by megablast (NCBI Blast 2.2.24). The mates of human genome aligned reads were re-aligned to the viral genome collection by megablast. Read-pairs that are aligned to viral genome collection and human genome, respectively, are suggestive of viral integration events. Those were extracted by a custom-made PERL script and annotated with RefSeq genes with BEDTools. The events were confirmed by visual inspection.

Supervised hierarchical clustering based on differentially expressed marker genes of eight defined molecular subgroups was used to minimize batch effects and allowed direct comparison of RNA-Seq data (Supplemental Table 5). Elevated LMO2 mRNA expression was detected in all cases and disregarded for hierarchical clustering analysis. Genes were regarded as marker genes for a molecular subgroup if at least eighty percent of the samples of the subgroup expressed the genes at a level within the top quartile of the whole cohort.

# Supplemental References

1. Liu Y, Easton J, Shao Y, Maciaszek J, Wang Z, Wilkinson MR*, et al.* The genomic landscape of pediatric and young adult T-lineage acute lymphoblastic leukemia. *Nature genetics* 2017 Jul 03.

2. Boztug K, Schmidt M, Schwarzer A, Banerjee PP, Diez IA, Dewey RA*, et al.* Stem-cell gene therapy for the Wiskott-Aldrich syndrome. *N Engl J Med* 2010 Nov 11; **363**(20)**:** 1918-1927.

3. Braun CJ, Boztug K, Paruzynski A, Witzel M, Schwarzer A, Rothe M*, et al.* Gene therapy for Wiskott-Aldrich syndrome--long-term efficacy and genotoxicity. *Sci Transl Med* 2014 Mar 12; **6**(227)**:** 227ra233.

4. Kordes M, Roring M, Heining C, Braun S, Hutter B, Richter D*, et al.* Cooperation of BRAF(F595L) and mutant HRAS in histiocytic sarcoma provides new insights into oncogenic BRAF signaling. *Leukemia* 2016 Apr; **30**(4)**:** 937-946.

5. Kleinheinz K, Bludau I, Huebschmann D, Heinold M, Kensche P, Gu Z*, et al.* ACEseq - allele specific copy number estimation from whole genome sequencing. 2017**:** 210807.

6. Wang J, Mullighan CG, Easton J, Roberts S, Heatley SL, Ma J*, et al.* CREST maps somatic structural variation in cancer genomes with base-pair resolution. *Nat Methods* 2011 Jun 12; **8**(8)**:** 652-654.

7. Quinlan AR, Hall IM. BEDTools: a flexible suite of utilities for comparing genomic features. *Bioinformatics* 2010 Mar 15; **26**(6)**:** 841-842.

8. Alexandrov LB, Nik-Zainal S, Wedge DC, Aparicio SA, Behjati S, Biankin AV*, et al.* Signatures of mutational processes in human cancer. *Nature* 2013 Aug 22; **500**(7463)**:** 415-421.

9. Ma X, Liu Y, Liu Y, Alexandrov LB, Edmonson MN, Gawad C*, et al.* Pan-cancer genome and transcriptome analyses of 1,699 paediatric leukaemias and solid tumours. *Nature* 2018 Mar 15; **555**(7696)**:** 371-376.
