# Supplementary material for: Comprehensive genomic characterization of gene therapy-induced T-cell acute lymphoblastic leukemia
Source: Leukemia. 2020 Mar 3;34(10):2785–9. doi: 10.1038/s41375-020-0779-z (PMC8321895; doi:10.1038/s41375-020-0779-z)

A

	all SNVs	all Indels	functional SNVs	functional Indels	Subclonal SNVs/ Indels (MAF<0.3)	Time after GT (days)
**WAS1**	870	233	13	3	19%	1813
**WAS5**	1172	275	6	2	63%	1073
**WAS6**	153	185	1	1	(50%)	483
**WAS7**	442	287	5	1	0%	1105
**WAS8**	932	393	11	2	38%	792
**WAS10**	1509	79	18	2	20%	1364

B

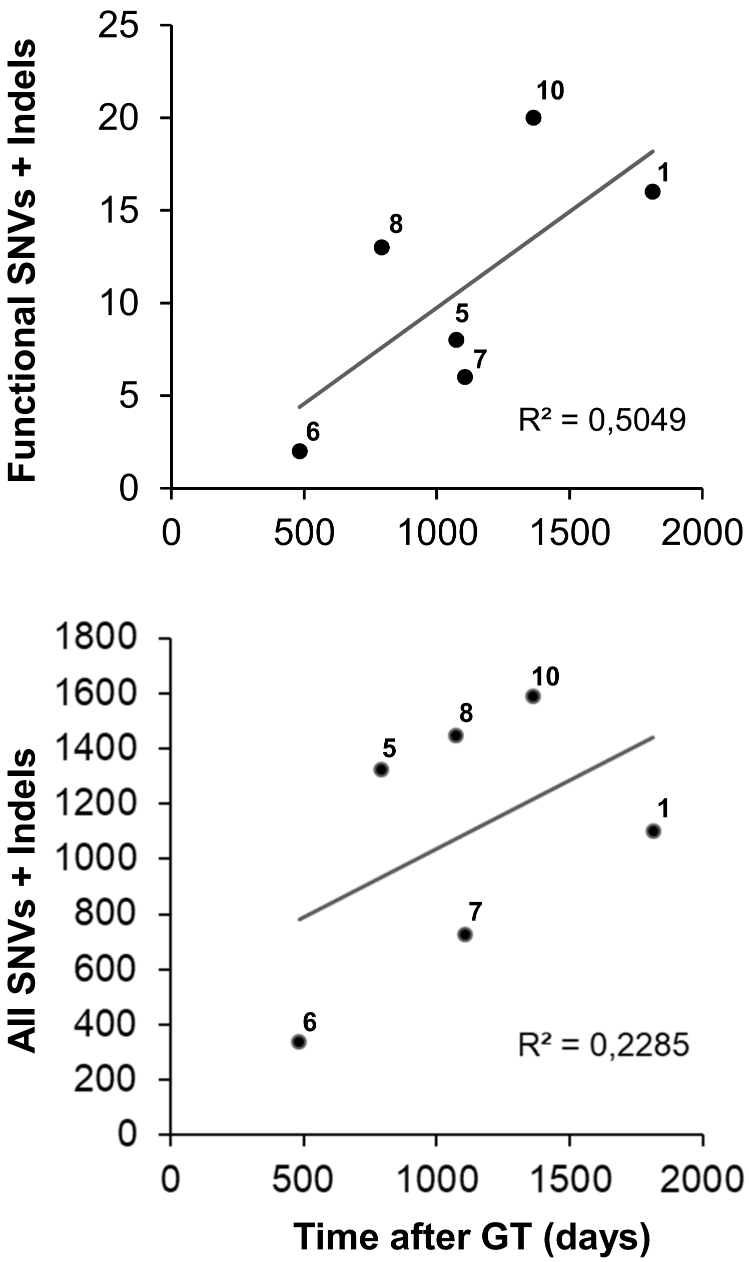

Supplement: Supplementary file 7 — Supplemental Figure 1 [file 41375_2020_779_MOESM7_ESM.pdf]
