# Supplementary figures and images for: Comprehensive genomic characterization of gene therapy-induced T-cell acute lymphoblastic leukemia
Source: Leukemia. 2020 Mar 3;34(10):2785–9. doi: 10.1038/s41375-020-0779-z (PMC8321895; doi:10.1038/s41375-020-0779-z)

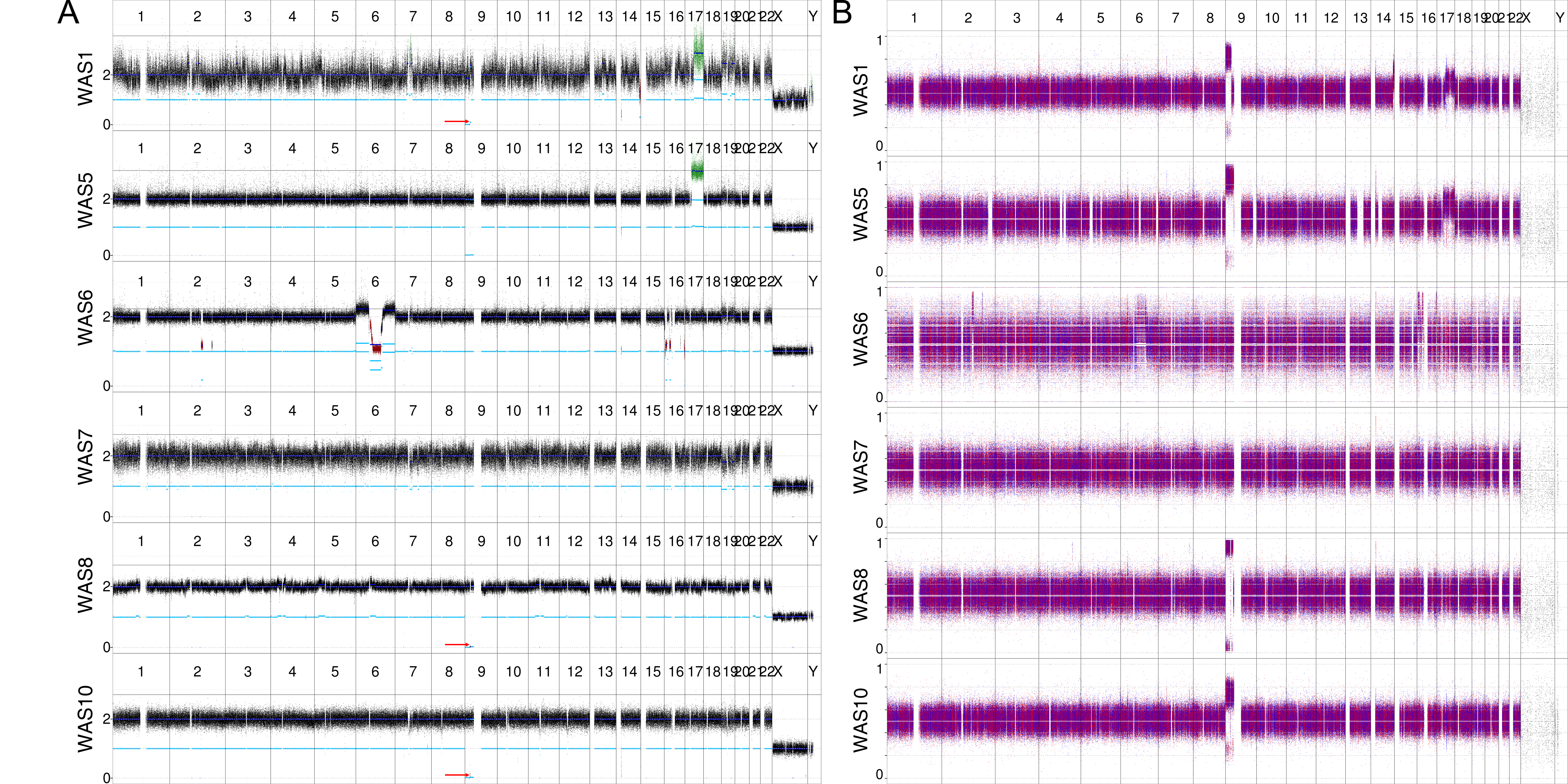

Supplement: Supplementary file 8 — Supplemental Figure 2 [file 41375_2020_779_MOESM8_ESM.tif]

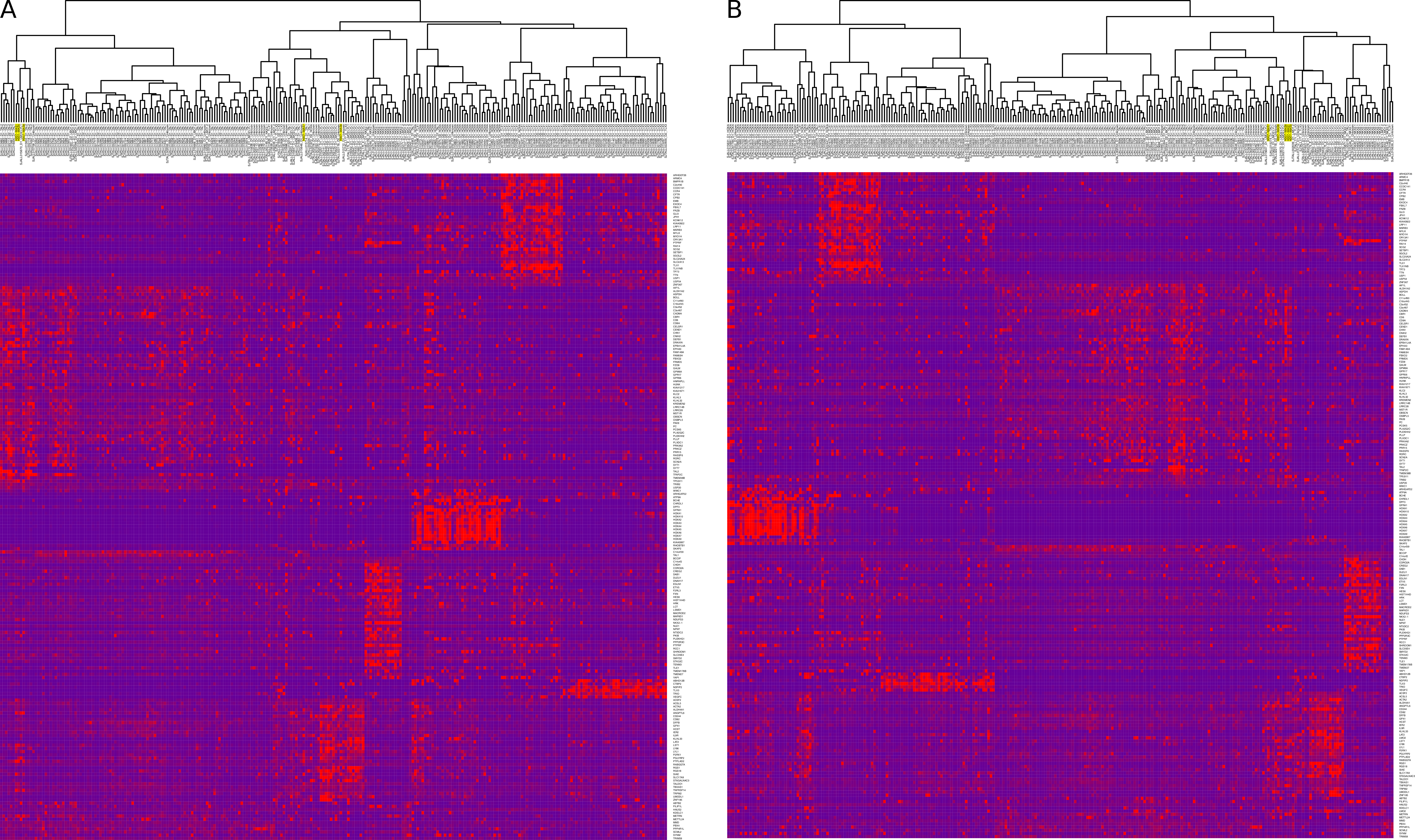

Supplement: Supplementary file 9 — Supplemental Figure 3 [file 41375_2020_779_MOESM9_ESM.tif]
