# Supplementary material for: Comprehensive genomic characterization of gene therapy-induced T-cell acute lymphoblastic leukemia
Source: Leukemia. 2020 Mar 3;34(10):2785–9. doi: 10.1038/s41375-020-0779-z (PMC8321895; doi:10.1038/s41375-020-0779-z)
Supplement: Supplementary file 10 — Supplemental Figure 4 [file 41375_2020_779_MOESM10_ESM.pdf]

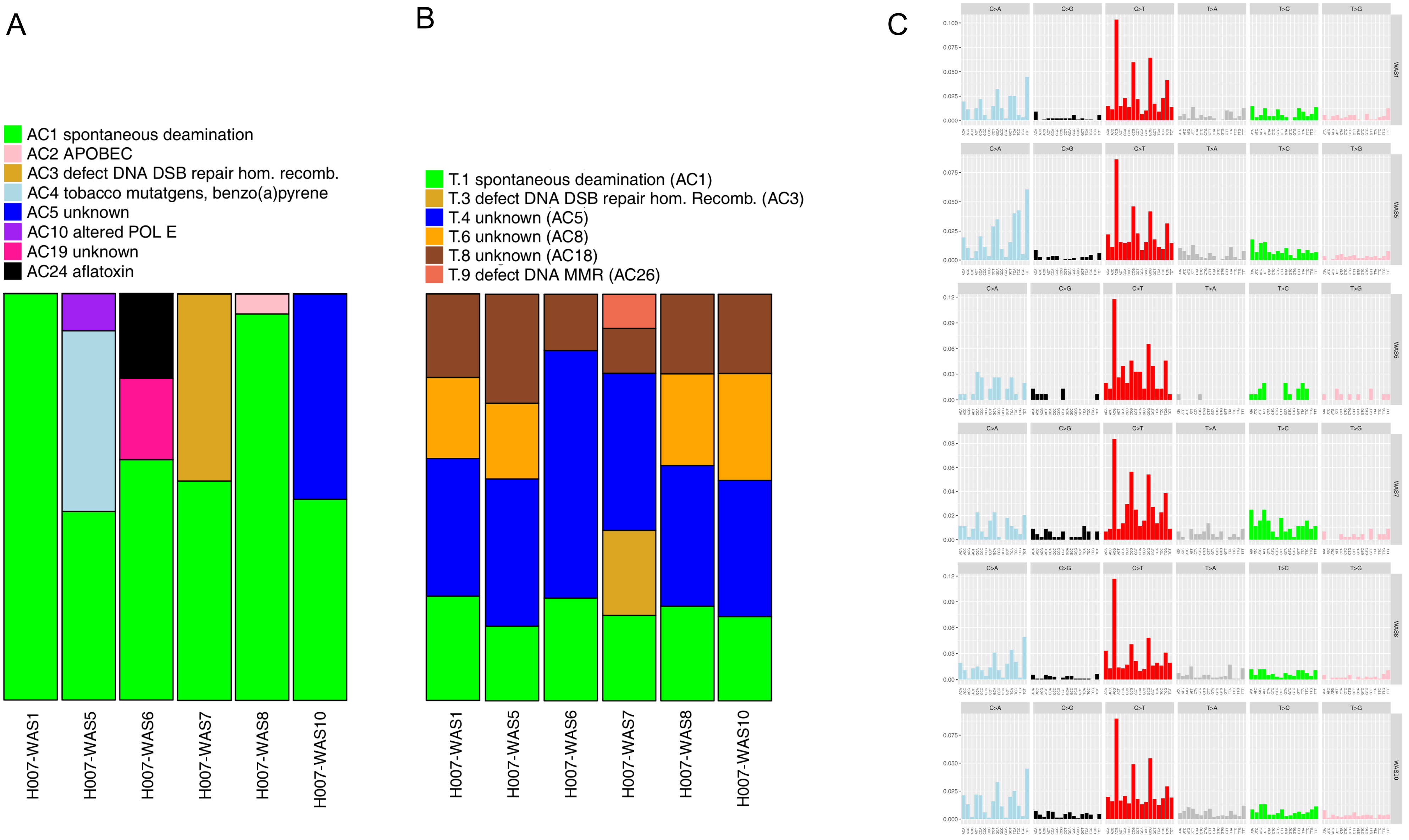
A
AC1 spontaneous deamination
AC2 APOBEC
AC3 defect DNA DSB repair hom. recomb.
AC4 tobacco mutatgens, benzo(a)pyrene
AC5 unknown
AC10 altered POL E
AC19 unknown
AC24 aflatoxin
H007-WAS1
H007-WAS5
H007-WAS6
H007-WAS7
H007-WAS8
H007-WAS10
B
T.1 spontaneous deamination (AC1)
T.3 defect DNA DSB repair hom. Recomb. (AC3)
T.4 unknown (AC5)
T.6 unknown (AC8)
T.8 unknown (AC18)
T.9 defect DNA MMR (AC26)
H007-WAS1
H007-WAS5
H007-WAS6
H007-WAS7
H007-WAS8
H007-WAS10
C
C>A
C>G
C>T
T>A
T>C
T>G
